# Serum exosomal miR-122 as a potential diagnostic and prognostic biomarker of colorectal cancer with liver metastasis

**DOI:** 10.7150/jca.33022

**Published:** 2020-01-01

**Authors:** Li Sun, Xiangxiang Liu, Bei Pan, Xiuxiu Hu, Yefei Zhu, Yingying Su, Zhirui Guo, Guoying Zhang, Mu Xu, Xueni Xu, Huiling Sun, Shukui Wang

**Affiliations:** 1 Department of General Clinical Research Center, Nanjing First Hospital, Nanjing Medical University, Nanjing, Jiangsu, China; 2 Department of Laboratory Medicine, the Second Affiliated Hospital, Nanjing Medical University, Nanjing, Jiangsu, China

**Keywords:** colorectal cancer, serum, exosomes, miRNA, diagnosis, prognosis.

## Abstract

**Background**: Liver is the most common site for metastatic spread of CRC at the time of diagnosis which leads to high mortality. This study aimed to identify novel circulating exosomal miRNAs as biomarkers of colorectal cancer (CRC) with liver metastasis (LM).

**Materials and methods**: Candidate miRNAs were selected through integrated analysis of Gene Expression Omnibus (GEO) database as well as clinical samples. Exosomes isolated from serum and cultured media were identified by using transmission electron microscopy (TEM) and western blot. The expression levels and diagnostic value of candidate miRNAs were further tested and validated through qRT-PCR and receiver operating characteristic curve (ROC) analysis. The association of candidate miRNA expressions with patients' prognosis was analyzed with logistic regression and Cox proportional hazards regression models.

**Results**: After integrated analysis of three GEO datasets and clinical samples, miR-122 was discovered to be remarkably overexpressed in tissues of CRC patients. Then we revealed that elevated serum miR-122 was tumor-derived by being packaged into exosomes. The expressions of serum exosomal miR-122 were significantly upregulated in CRC patients, especially in those with LM. Serum exosomal miR-122 expressions could differentiate CRC patients with LM from healthy controls and patients without LM with area under the ROC curve (AUC) of 0.89 and 0.81. Uni- and multivariate logistic regression showed that serum exosomal miR-122 was an independent prognostic indicator of CRC patients.

**Conclusions:** Serum exosomal miR-122 was a novel potential diagnostic and prognostic biomarker in CRC patients with LM.

## Introduction

Colorectal cancer (CRC), one of the most common cancers, is a major cause of cancer-related deaths worldwide 1. The survival rates of CRC patients have increased in recent years somewhat due to earlier diagnosis as well as advanced treatment strategies 2, 3. However, approximately 20 - 25% of CRC patients have underwent liver metastasis (LM) which is the most common type for metastatic spread of CRC at the time of diagnosis 4, 5. CRC patients with LM usually receive intensive chemotherapy in combination with monoclonal antibodies therapy 6. Without a screening of CRC patients with LM, overtreatment with these extremely toxic and expensive agents not only aggravates the financial burden of patients, but also produces severe side-effects 7. Therefore, in order to realize personalized treatment strategies for CRC patients, novel biomarkers, particularly with non-invasion, for the detection of CRC patients with LM are urgently needed.

Currently, serum-based tumor biomarkers have been widely accepted, such as carcinoembryonic antibody (CEA) 8. Unfortunately, except for neither sensitive nor specific for diagnosing CRC, CEA levels are not always correlated with the presence of metastasis 9. Accumulating studies indicates that circulating microRNAs (miRNAs) are promising surrogate minimally invasive biomarkers due to their ability of resisting to endogenous ribonuclease activity, extreme pH and temperature 10. miRNAs, about 22 nucleotides, are a class of short single-stranded non-coding RNAs which cause target mRNA molecules either degradation or translational inhibition by binding to the 3' untranslated region (UTR) of mRNAs 11. Indeed, several studies have reported the value of circulating miRNAs in detecting cancer patients with metastasis. Wu et al. indicated that circulating miR-422a is associated with lymphatic metastasis in lung cancer 12. Guo et al. declared that serum miR-21 serves as a biomarker for hepatocellular carcinoma with distant metastasis 13. Chen and colleagues identified plasma miR-122 and miR-192 as potential novel biomarkers for the early detection of distant metastasis of gastric cancer 14. In CRC, in spite of several studies reporting circulating miRNAs are significantly associated with metastasis of CRC 15, 16, the diagnostic utility of circulating miRNAs reminds elusive. Besides, the origin of these miRNAs has not been clarified yet.

Circulating exosomes are small membrane vesicles (30-150 nm) that are released into the extracellular environment upon fusion of multivesicular bodies with cellular membrane 17. These vesicles, loaded with proteins and unique RNAs, have a wide range of biological functions, such as cell-to-cell communication 18. Our previous study showed that circulating exosomal miR-27a and miR-130a were novel diagnostic and prognostic biomarkers of CRC 19. However, specific miRNAs in serum exosome associated with LM have not been adequately investigated in CRC.

In this study, after integrated analysis of three GEO datasets and clinical samples, we found miR-122 was significantly overexpressed in CRC patients, especially in those with LM. Thereafter, we discovered that elevated serum miR-122 in CRC patients was delivered by exosomes and released by tumor. Subsequently, we explored the diagnostic and prognostic utility of serum exosomal miR-122. Our results showed that serum exosomal miR-122 could obviously discriminate CRC patients with LM from healthy individuals as well as CRC patients without LM. Besides, CRC patients with higher circulating exosomal miR-122 expression suffered from unfavorable prognosis.

## Materials and methods

### Patients

All CRC patients were enrolled from Nanjing First Hospital. Patients were confirmed through histopathological analysis of surgical resected tumor. Gender and age matched healthy subjects were collected from those who participate in the physical examination. Written informed consent was obtained from all patients and healthy individuals. This study was approved by the Research and Ethical Committee of Nanjing First Hospital.

### Samples Processing

After being centrifuged at 4000rpm for 10min, serum samples collected from venous blood were stored at -80 ℃ until further analysis. Tissue samples were frozen in liquid nitrogen immediately after being surgical resected. Total RNA was isolated using Trizol LS reagent (Invitrogen, USA) according to the manufacturer's protocol. Cel-miR-39-3p (Takara, Japan), act as external reference, was added into each sample at a concentration of 1uMol/L.

### Analysis of GEO database

GEO datasets about miRNA expression in CRC metastasis were searched in GEO database using keywords “microRNA”, “colorectal cancer”, and “metastasis”. The differentially expressed miRNAs were analyzed by using online tool GEO2R.

### Cell cultures

The normal colonic mucosal epithelial cell (FHC) along with CRC cell lines (HCT8, HT29, HCT116, SW480, and SW620) were purchased from American Type Culture Collection (Manassas, VA, USA) and had been tested and authenticated through STR (Short Tandem Repeat) method. Cells were cultured in Dulbecco's Modifed Eagle's Medium (DMEM) supplemented with 100 µl fetal bovine serum (FBS) (Gibco, Vienna, Austria), 10 µl penicillin (Gibco, Vienna, Austria) and 10 µl streptomycin (Gibco, Vienna, Austria) per ml medium in humidified atmosphere containing 5% CO^2^ at 37˚C. When growing at 80% confluency, SW620 cells were treated with 10 uM GW4869 (Sigma, USA), an exosomes inhibitor, for 2 h.

### Exosomes purification

Exosomes were isolated from cultured media or serum by using Exosome Isolation Reagent according to the manufacturer's instruction (Invitrogen, USA). Briefly, after collecting media (serum), cellular debris were removed by centrifugation at 2000 × g for 30 min. Then medium (serum) was transferred to a fresh tube and exosomes were precipitated using precipitation solution at 4 °C overnight. The mixture was centrifuged at 10000 × g for 60 min to pellet exosomes. Finally, exosomes were resuspended in PBS.

### Transmission electron microscopy analysis

Isolated exosomes were fixed in 2.5% glutaraldehyde solution for at least 2 hours. Next, 10 μL of the diluted mixtures were transferred to a cleaned copper net. Images were obtained by TEM (JEM-1010, Jeol, Japan) after dyeing with 2% phosphotungstic acid solution.

### Western blot

Briefly, exosomes isolated were lysed to harvest protein. Denatured protein was separated by 10% SDS-PAGE, transferred to PVDF membranes. The membranes were blocked with TBST that contains 5% skimmed milk, then blocked with primary antibodies (mouse anti-CD63, 1:1000, ab59479; mouse anti-TSG101, 1:1000, ab83) overnight at 4℃. After being washed four times, the membranes were incubated with anti-IgG conjugated to horseradish peroxidase at room temperature for 1h. Bands were visualized using the enhanced chemiluminescence system (ECL) reagent (KeyGEN BioTECH, China).

### Quantitative real-time PCR for miRNA quantitation

Reverse transcription and qRT-PCR for miR-122, external reference miR-39 and endogenous control U6 snRNA were performed using Hairpin-it^TM^ microRNA RT-PCR Quantitation Kit (GenePharma, China) according the manufacture's instructions. The reactions were initiated with denaturation at 95℃ for 3min, followed by 40 cycles of 95℃ for 15s and 62℃ for 34s. The relative expression of miR-122 was calculated by 2^ -△△Ct^ method. △Ct=Ct_miRNA_-Ct_miR-39/U6._

### Statistical analysis

Statistical analysis of the differences between groups were performed by SPSS 19.0 (IBM, USA) or GraphPad Prism 5.0 (GraphPad Software, USA) using the Student's paired or unpaired t-test or one-way ANOVA. The ROC was performed to evaluate the utility of serum exosomal miR-122 as a diagnostic biomarker of CRC with LM. Cutoff value of the expression of miR-122 was determined by Youden index. The association of serum exosomal miR-122 levels with patients' survival was analyzed by using univariate and multivariate logistic regression and Cox proportional hazards regression models. P value < 0.05 was considered to be statistically significant.

## Results

### MiR-122 expressions were remarkably elevated in CRC with LM

**Figure 1:**
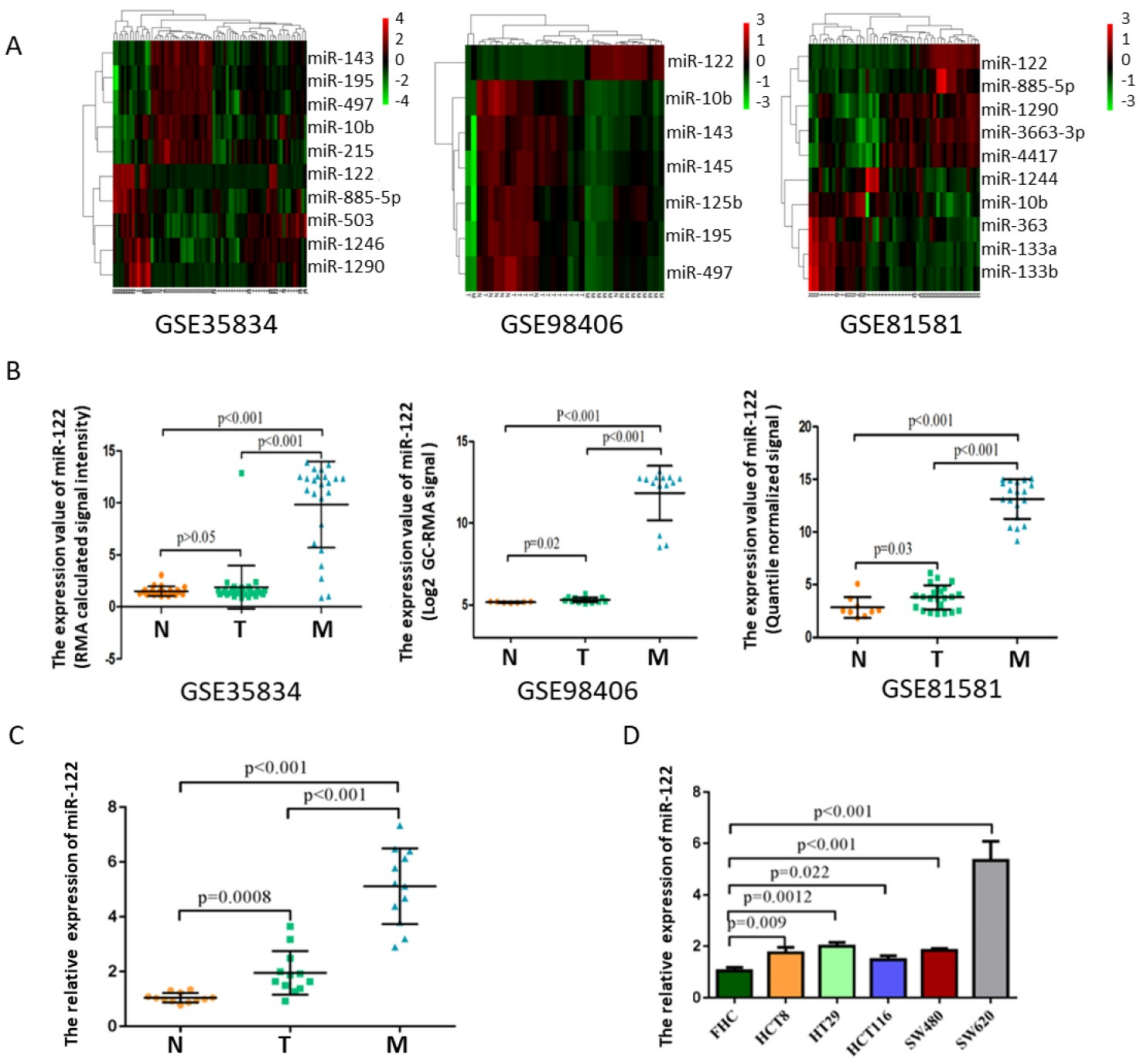
MiR-122 expressions were remarkably upregulated in CRC with LM. **(A)** Hotmap of representative miRNAs significantly deregulated in CRC with LM based on GEO datasets. **(B)** The expression value of miR-122 in three GEO datasets. **(C-D)** The expression levels of miR-122 were significantly upregulated in CRC tissues and cells.

After manually searching in GEO database, three datasets (GSE35834, GSE81581, GSE98406) which conducted a comparative analysis of miRNA expression profiles in colorectal liver metastasis (M), primary colorectal tumor (T) and normal colorectal mucosa (N) were selected. Among these deregulated miRNAs with ∣logFC∣>1 and p<0.05, miR-122 expressions showed consistently highest differential fold-change in tissues of colorectal liver metastasis (Figure 1A-B). The expression levels of tissular miR-122 were validated in a small set of colorectal liver metastasis (n=12), primary colorectal tumor (n=12) and normal colorectal mucosa (n=12). MiR-122 expressions were significantly elevated in colorectal liver metastasis when compared to primary colorectal tumor and normal colorectal mucosa (Figure 1C). Besides, we investigated the cellular expression of miR-122 in human CRC cell lines. As shown in Figure 1D, the expressions of miR-122 were significantly higher in CRC cell lines, especially in SW620 cells. Mounting evidences have illustrated that tumor cells could package miRNAs into exosomes and release it into peripheral blood subsequently to promote the migration of tumor 20-22. Maierthaler et al. reported that plasma miR-122 was significantly upregulated in CRC patients 23. We wondered whether elevated miR-122 in peripheral blood of CRC patients were tumor-derived through being delivered by exosomes and whether circulating exosomal miR-122 was a potential diagnostic and prognostic biomarker of CRC with LM.

### Elevated serum miR-122 were tumor-derived through being packaged into exosomes

**Figure 2:**
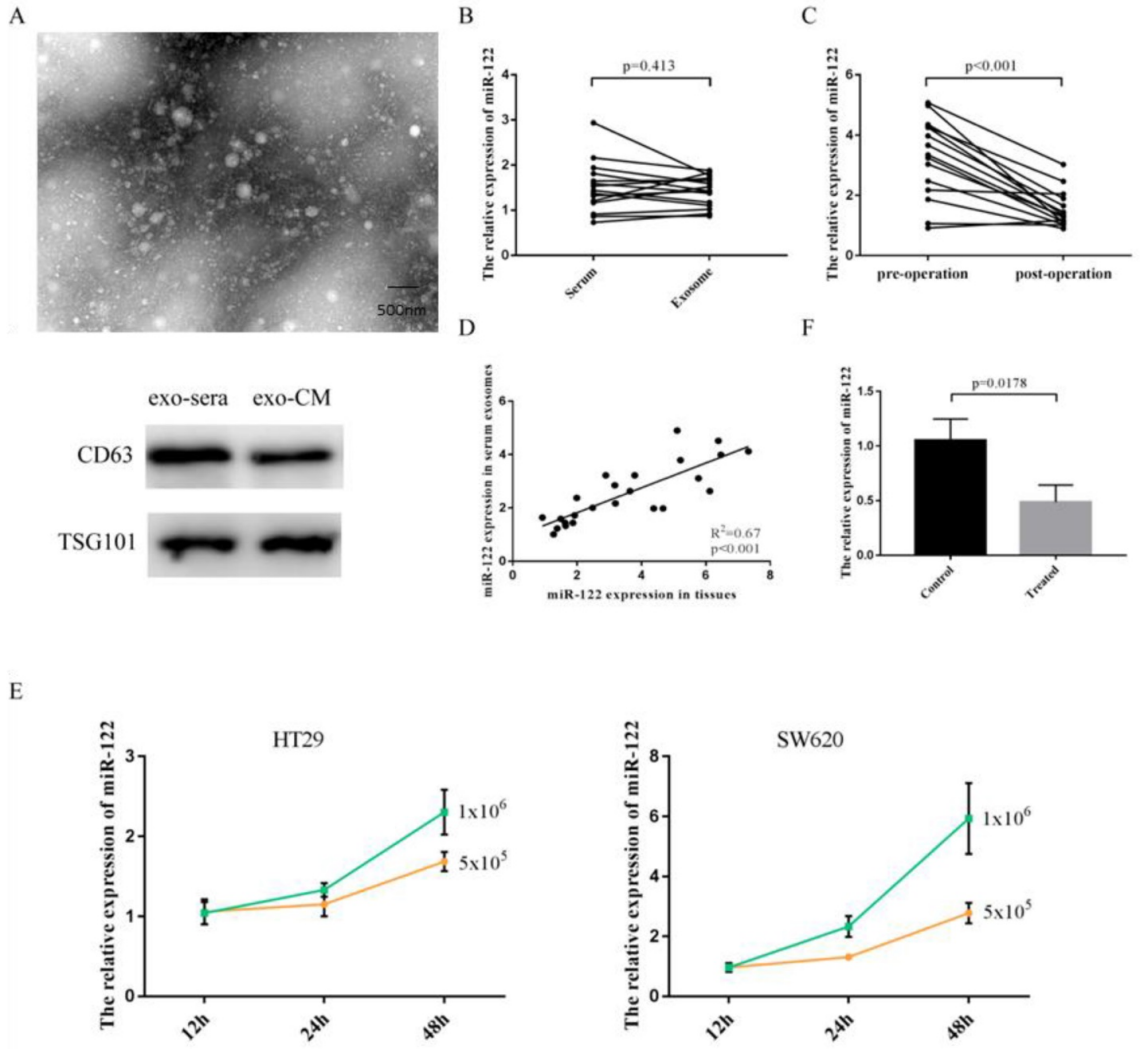
Elevated serum miR-122 was tumor-derived. **(A)** Exosomes were identified using TEM and western blot. **(B)** The expressions of miR-122 in serum did not differ from that in pure exosomes isolated from equivolumetric serum. **(C)** The expressions of serum exosomal miR-122 were significantly downregulated after tumor being resected. **(D)** The expressions of exosomal miR-122 in serum of CRC patients were positively correlated with that in CRC tissues. **(E)** Exosomal miR-122 expressions in the culture media from both cell lines increased with time and with increasing numbers of cells. **(F)** The expressions of exosomal miR-122 in the culture media were significantly downregulated after being treated with GW4869.

Exosomes isolated from serum and cultured media were identified by using transmission electron microscope (TEM) and specific protein markers (Figure 2A). As shown in Figure 2B, the expressions of miR-122 in serum did not differ from that in pure exosomes isolated from equivolumetric serum. After tumor being resected, the expressions of serum exosomal miR-122 were significantly downregulated (Figure 2C). Furthermore, we discovered that the expressions of exosomal miR-122 in serum of CRC patients were positively correlated with that in CRC tissues (Figure 2D). Next, we confirmed the secretory potential of exosomal miR-122 into the culture media by using HT29 and SW620 cells. We observed that exosomal miR-122 expressions in the culture media from both cell lines increased with time and with increasing numbers of cells (Figure 2E). Besides, the expressions of exosomal miR-122 in the culture media which were treated with GW4869 were significantly downregulated compared to negative controls (Figure 2F).

### High expressions of serum exosomal miR-122 in CRC patients with LM explored in the training step

**Figure 3:**
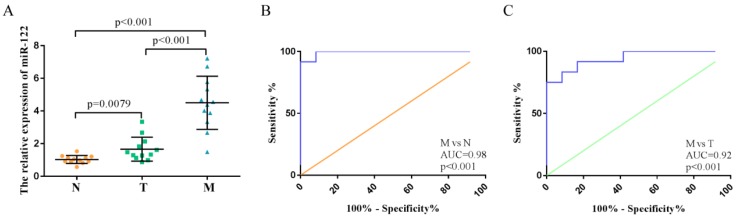
High expressions of serum exosomal miR-122 in CRC patients confirmed in a small set of subjects. **(A)** The relative expression levels of exosomal miR-122 were significantly upregulated in the serums of CRC patients with liver metastasis. **(B)** The diagnostic utility of serum exosomal miR-122 to differentiate CRC patients with LM from healthy subjects. **(C)** The diagnostic utility of serum exosomal miR-122 to differentiate CRC patients with LM from CRC patients without LM.

We investigated the expression levels of exosomal miR-122 in serum samples from 36 subjects with colorectal liver metastasis (n=12), primary colorectal tumor (n=12) and normal colorectal mucosa (n=12). MiR-122 expression levels in the serum exosome of CRC patients with LM were significantly higher compared to those from normal subjects and CRC patients without LM (Figure 3A). ROC analyses showed that serum exosomal miR-122 could differentiate CRC patients with LM from healthy controls and CRC patients without LM with an AUC value of 0.98 (95% CI:0.93-1.0, p<0.001) and 0.92 (95% CI:0.85-0.99, p<0.001), respectively (Figure 3B-C).

### The expression levels and diagnostic role of serum exosomal miR-122 validated in an independent cohort

**Figure 4:**
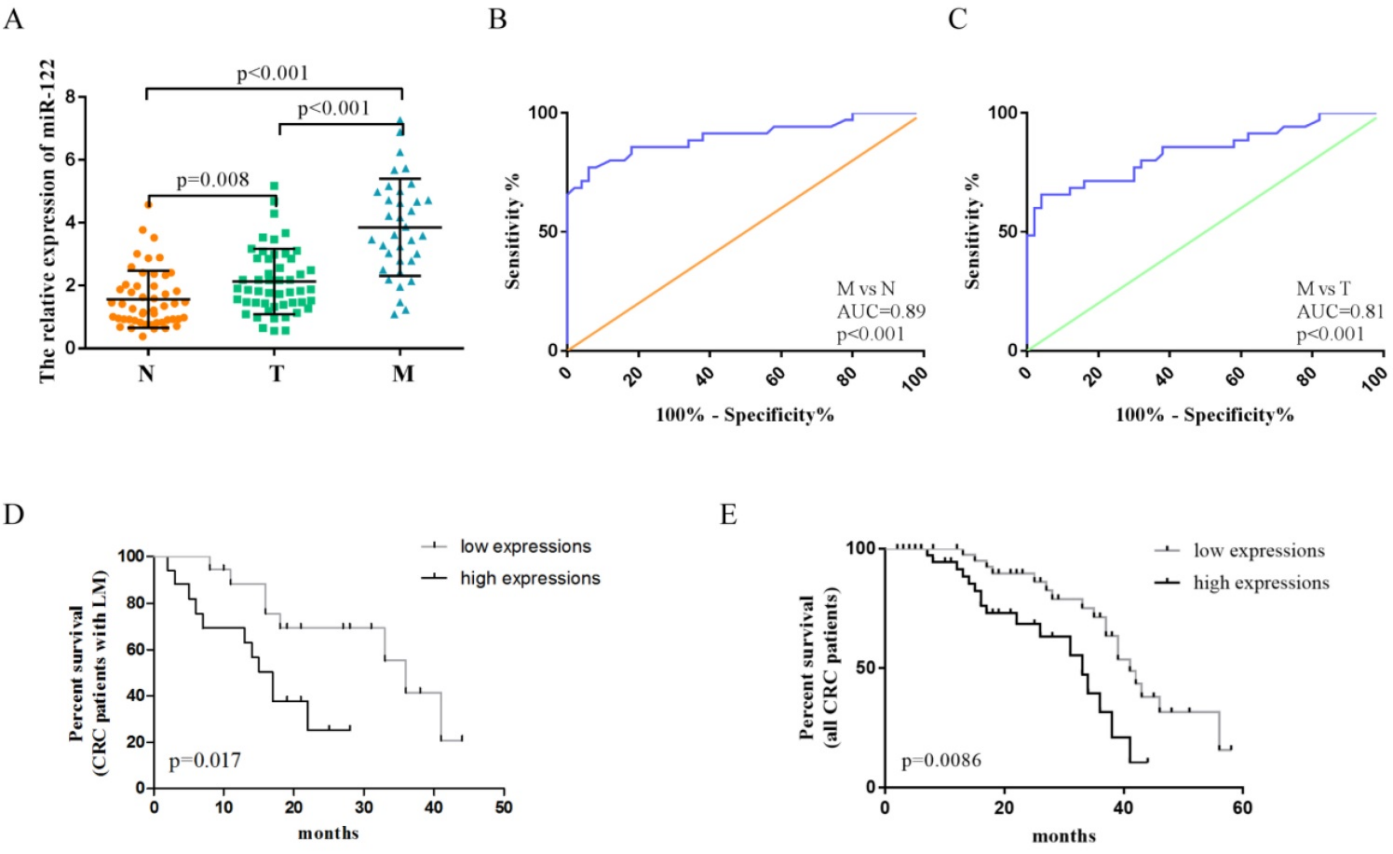
The diagnostic value of circulating exosomal miR-122 validated in an independent cohort. **(A)** The expression levels of serum exosomal miR-122 were significantly upregulated in CRC patients with/without LM. **(B)** The diagnostic utility of serum exosomal miR-122 to differentiate CRC patients with LM from healthy subjects. **(C)** The diagnostic utility of serum exosomal miR-122 to differentiate CRC patients with LM from CRC patients without LM. **(D-E)** CRC patients, especially those with LM, who have high serum exosomal miR-122 expressions may suffer from poor OS.

The clinicopathological characteristics of CRC patients in the validation phase were summarized in Table 1. A total of 135 serum samples, including those from CRC patients with LM (n= 35), CRC patients without LM (n =50), and healthy controls (n= 50), were examined to evaluate the diagnostic potential of serum exosomal miR-122. Serum exosomal miR-122 expression levels were significantly elevated in CRC patients, especially in patients with LM, when compared to healthy controls (Figure 4A). In addition, serum exosomal miR-122 levels were significantly higher in CRC patients with larger tumor size (p=0.0069), advanced TNM stage (p=0.0192), and liver metastasis (p<0.001). ROC analyses showed that serum exosomal miR-122 could differentiate CRC patients with LM from healthy controls and CRC patients without LM with an AUC value of 0.89 (95% CI: 0.83-0.95, p<0.001) and 0.81 (95% CI: 0.72-0.90, p<0.001), respectively (Figure 4B-C).

### Serum exosomal miR-122 was a prognostic indicator of CRC patients

Plasma miR-122 was reported to be a potential prognostic indicator in human glioma 24. Since elevated serum exosomal miR-122 were tumor-derived, we wondered whether exosomal miR-122 in peripheral blood could predict prognosis of CRC patients. The expression levels of exosomal miR-122 in sera of CRC patients were categorized as high (n = 42) or low (n = 43) according to the median value (n-2.5). Next, Kaplan-Meier survival analysis was used to explore the association of serum exosomal miR-122 expressions and clinical outcomes of CRC patients. The result showed that CRC patients, especially those with LM, who have high serum exosomal miR-122 expressions may suffer from poor OS (Figure 4D-E). Univariate analysis revealed that serum exosomal miR-122 expressions, TNM stage, lymph node metastasis as well as liver metastasis status were significantly associated with the OS of CRC patients. Multivariate analysis indicated that serum exosomal miR-122 expressions and liver metastasis status were independent prognostic factors for the OS of CRC patients (Table ​2).

## Discussion

In this study, we were the first to discover that elevated serum miR-122 in CRC patients was delivered by exosomes and released by tumor. Moreover, we identified serum exosomal miR-122 as a specific diagnostic marker of CRC with LM because serum exosomal miR-122 expressions could obviously discriminate CRC patients with LM from healthy individuals as well as CRC patients without LM. Finally, we found that CRC patients with higher circulating exosomal miR-122 expression suffered from unfavorable prognosis.

Circulating miRNAs serving as biomarkers of CRC have been widely reported. Liu et al. observed miR-1260b is a potential prognostic biomarker of CRC 25. Imaoka et al. reported circulating miR-1290 as a novel diagnostic and prognostic biomarker in human CRC 26. Ma et al. documented miR-150 as a potential biomarker associated with prognosis and therapeutic outcome in CRC 27. However, circulating miRNAs acting as specific biomarkers for detecting CRC with metastasis have not been clarified. In recent decades, increasing studies have focused on exosomes, a subclass of extracellular vesicles, which were involved in intercellular communication and released by most cell types 28. Cells can trigger cancer-related disorders after the recognition and uptake of circulating exosomal miRNAs, providing indications for early tumor biopsy, diagnosis, and treatment 29, 30. Here, we demenstrated that serum exosomal miR-122 expressions were tumor-derived and remarkably upregulated in CRC patients with LM. The circulating exosomal miR-122 levels could differentiate CRC patients with LM from CRC patients without LM and healthy controls with high sensitivity and specificity. However, the sample size included in this study was relatively small, and we believe that prospective studies with larger sample numbers are required to clarify the usefulness of serum exosomal miR-122 as a novel diagnostic biomarker of CRC with LM. Chen et al. exhibited circulating miR-122 as a potential novel biomarker for the detection of distant metastasis of gastric cancer 14. Therefore, whether serum miR-122 could serve as a biomarker for detecting distant metastasis of whole gastroenteric cancer needed to be further explored. Besides, based on study of Maierthaler et al. 23, our study not only illustrated the reason of circulating miR-122 as a prognostic factor of CRC, but also demonstrated exosomal miR-122 in peripheral blood could also predict the prognosis of CRC patients.

In conclusion, we demonstrated that serum exosomal miR-122 was a novel potential diagnostic and prognostic biomarker of CRC patients with LM.

## Figures and Tables

**Table 1:** Correlations between serum exosomal miR-122 expression levels and clinicopathological features in CRC patients.

Variables	Serum exosomal miR-122 expressions		
	Number	Mean±SD	P value
Age			
<65	28	3.016 ± 0.267	P=0.695
≥65	57	2.867 ± 0.233	
Gender			
Male	46	2.737 ± 0.2136	P=0.875
Female	39	2.786 ± 0.2053	
Histology differentiation			
Well/moderate	57	2.280 ± 0.2022	P=0.274
Poor	28	2.669 ± 0.2310	
Tumor size			
<4 cm	54	1.577 ± 0.2108	P=0.0069
≥4 cm	31	2.867 ± 0.1379	
TNM stage			
I/II	47	2.216 ± 0.217	P=0.0192
III/IV	38	3.252 ± 0.166	
Lymph node metastasis			
Negative	48	2.223 ± 0.1357	P=0.0744
Positive	37	2.694 ± 0.1419	
Liver metastasis			
Negative	50	1.199 ± 0.08	P<0.001
Positive	35	4.039 ± 0.345	

**Table 2:** Univariate and multivariate analysis for OS of CRC patients.

Variables	univariate analysis			multivariate analysis		
	HR	95%CI	P value	HR	95%CI	P value
Age (≥65, <65)	0.91	0.63-1.46	0.52			
Gender (male,female)	0.87	0.53-1.31	0.37			
Histology differentiation (poor, moderate+well)	1.12	0.87-2.04	0.17			
Tumor size (≥4 cm, <4 cm)	1.27	0.79-2.28	0.29			
TNM stage (III/IV, I/II)	1.35	1.04-4.73	0.032	1.21	0.93-3.01	0.097
Lymph node metastasis (postive, negative)	1.92	1.09-3.56	0.014	1.76	0.98-3.61	0.057
Liver metastasis (postive, negative)	3.52	1.73-6.14	<0.001	3.83	1.92-6.64	<0.001
miR-122 expressions (high,low)	2.39	1.15-4.99	0.0086	1.69	1.08-3.77	0.012
